# Arenavirus Budding

**DOI:** 10.1155/2011/180326

**Published:** 2011-09-08

**Authors:** Shuzo Urata, Juan Carlos de la Torre

**Affiliations:** ^1^Department of Emerging Infectious Disease, Institute of Tropical Medicine, Nagasaki University, 1-12-4 Sakamoto, Nagasaki 852-8523, Japan; ^2^Department of Immunology and Microbial Science, The Scripps Research Institute, 10550 North Torrey Pines Road, La Jolla, CA 92037, USA

## Abstract

Several arenaviruses cause hemorrhagic fever disease in humans and pose a significant public health concern in their endemic regions. On the other hand, the prototypic arenavirus LCMV is a superb workhorse for the investigation of virus-host interactions and associated disease. The arenavirus small RING finger protein called Z has been shown to be the main driving force of virus budding. The budding activity of Z is mediated by late (L) domain motifs, PT/SAP, and PPXY, located at the C-terminus of Z. This paper will present the current knowledge on arenavirus budding including the diversity of L domain motifs used by different arenaviruses. We will also discuss how improved knowledge of arenavirus budding may facilitate the development of novel antiviral strategies to combat human pathogenic arenaviruses.

## 1. Introduction

Arenaviruses are enveloped viruses with a bisegmented negative strand (NS) RNA genome with coding capability for four known genes: nucleoprotein (NP), surface glycoprotein precursor (GPC), polymerase (L), and matrix-like (Z) proteins. Despite their limited genome and proteomic complexity, arenaviruses are able to exhibit very different phenotypic infection outcomes ranging from long-term subclinical chronic infections on their natural rodent hosts [1] to hemorrhagic fever (HF) disease in humans, infected through mucosal exposure to aerosols or by direct contact of abrade skin with infectious material. Thus, Lassa virus (LASV), the causative agent of Lassa fever (LF) is estimated to infect several hundred thousand individuals yearly in its endemic regions of West Africa, resulting in a high number of LF cases associated with high morbidity and significant mortality. Likewise, Junin virus (JUNV) causes Argentine HF, a severe illness with hemorrhagic and neurological manifestations and a case fatality of 15–30%, whereas the Machupo (MACV) and Guanarito (GTOV) arenaviruses emerged as causative agents of HF in Bolivia and Venezuela, respectively. On the other hand, the prototypic arenavirus, lymphocytic choriomeningitis virus (LCMV), is a superb workhorse for the investigation of virus-host interactions including mechanisms of virus control and clearance by the host immune defenses, as well as viral counteracting measures leading to chronic infection and associated disease [2, 3]. Moreover, evidence indicates that the globally distributed prototypic arenavirus LCMV is a neglected human pathogen of clinical significance, especially in cases of congenital infection. In addition, LCMV poses a special threat to immunocompromised individuals, as illustrated by cases of transplant-associated infections by LCMV with a fatal outcome in the USA and Australia. Public health concerns about arenavirus infections are aggravated by the lack of licensed vaccines and current therapy being limited to the use of the nucleoside analog ribavirin, which is only partially effective, requires early and intravenous administration for optimal activity, and can cause significant side effects. Therefore, it is important to develop novel and effective antiarenaviral strategies, a task that should be facilitated by a better understanding of the arenavirus molecular and cell biology. 

The arenavirus small RING finger Z protein has been shown to be the main driving force of budding. This paper will examine our current understanding of arenavirus budding and discuss potential implications for the development of novel targeting strategies to combat human pathogenic arenaviruses.

## 2. Arenavirus Genome Organization and Life Cycle

**Figure 1:**
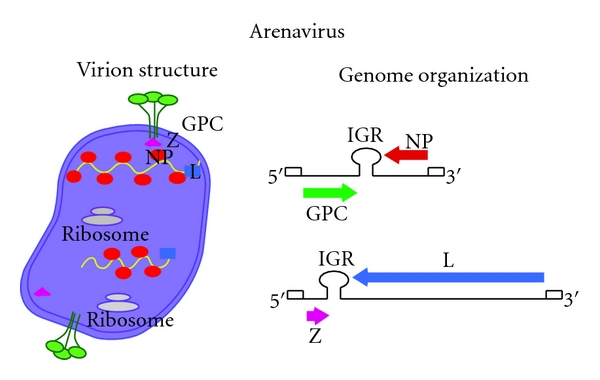
Arenavirus virion structure and genome organization. Arenaviruses are enveloped viruses with a bisegmented negative strand RNA genome. Each genome segment uses an ambisense-coding strategy to direct the synthesis of two viral polypeptides. The S (ca 3.5 kb) segment encodes for the viral nucleoprotein (NP) and glycoprotein precursor (GPC). GPC is posttranslational processed by the cellular protease S1P into the mature virion surface GP1 and GP2. The L (ca 7.3 kb) segment encodes for the virus RNA-dependent RNA polymerase (L) and a small RING finger protein (Z) that is functionally the arenavirus counterpart of the matrix (M) protein found in many enveloped negative strand RNA viruses. IGR, noncoding intergenic region.

Arenaviruses are enveloped viruses with a bisegmented negative strand (NS) RNA genome and a life cycle restricted to the cell cytoplasm. Virions are pleomorphic but often spherical and covered with surface glycoprotein spikes. Both the large, L (ca 7.3 kb) and small, S (ca 3.5 kb) genome RNA species use an ambisense-coding strategy to direct the synthesis of two polypeptides in opposite orientation, separated by a noncoding intergenic region (IGR) with a predicted folding of a stable hairpin structure [1] (Figure 1). The S RNA encodes the viral glycoprotein precursor, GPC, (ca 75 kDa) and the nucleoprotein, NP, (ca 63 kDa), whereas the L RNA encodes the viral RNA-dependent RNA polymerase (RdRp, or L polymerase) (ca 200 kDa) and a small (ca 11 kDa) RING finger protein Z that is functionally the counterpart of the matrix (M) protein found in many enveloped NS RNA viruses. 

Consistent with a broad host range and cell-type tropism, a highly conserved and widely expressed cell surface protein *α*-Dystroglycan (*α*-DG) has been identified as a main receptor for LCMV, LASV, and several other arenaviruses [4, 5], whereas the human transferrin receptor (TfR) was identified as the primary receptor used by several New World (NW) arenavirus [6]. Upon receptor binding, virions are internalized using an endocytotic pathway that is either clathrin-independent or clathrin-dependent for Old World (OW) and NW arenavirus, respectively [5]. Interestingly, cell entry of OW LCMV and LASV are independent of caveolin, dynamin, actin, or small GTPases Rab5 and Rab7 but cholesterol-dependent [7–9]. Following the release of the viral ribonucleoprotein into the cytoplasm of the infected cells, the associated polymerase directs the biosynthetic processes involved in RNA replication and gene transcription. Assembly and cell release of infectious progeny involve the association of the viral ribonucleoprotein core with the surface GP complex, a process that is required for the production of infectious virions, which bud from the plasma membrane (PM).

## 3. Arenavirus Z Structure and Function

Results derived from minigenome- (MG-) based assays identified NP and L as the minimal viral transacting factors required for efficient RNA synthesis mediated by the virus polymerase [10–12]. Z was not required for RNA replication or transcription, but rather Z has been shown to exhibit a dose-dependent inhibitory effect on both transcription and replication of LCMV, Tacaribe virus (TACV), and LASV MGs [10, 12–14]. The inhibitory activity of Z on RNA synthesis by the LCMV polymerase did not require the N-terminus or C-terminus of Z, whereas the RING domain was strictly required but not sufficient [13, 14]. RING domains are known to mediate protein-protein interactions, and Z protein has been documented to interact with a variety of host cellular proteins including PML [15, 16] and translation initiation factor eIF4E [15–18]. The Z-PML interaction was reported to result in disruption of PML nuclear bodies and redistribution of PML to the cytoplasm, but the biological implications of this remain to be determined. On the other hand the Z-eIF4E interaction was found to impair eIF4E-dependent translation through its RING domain [16]. Interestingly, expression of Interferon regulatory factor 7 (IRF7), a key factor in the regulation of type I interferon (IFN) production by pDCs, is highly dependent on 4E [19]. Therefore, it is plausible that Z might mediate inhibition of IRF7 expression in arenavirus-infected pDCs and, thus, contributing to the mechanisms by which arenaviruses overcome the innate immune response by the host. 

The possible contribution of RING-mediated Z-host cellular protein interactions to arenavirus budding is currently unknown. Notably, arenavirus Z proteins have a strictly conserved W residue in proximity to the second conserved C residue within the RING, a feature characteristic of RING proteins with E3 ligase activity involved in ubiquitin-dependent protein degradation. However, preliminary evidence indicated that LASV Z protein lacked ubiquitin-ligating activity in the presence of a variety of E2 enzymes including Ubc4 and Cdc34/Ubc3 [16]. Whether arenavirus Z proteins may exhibit E3 ligase activity in the presence of other E2 ubiquitin-conjugating enzymes and their biological implications remain to be determined. Z has also been implicated in antagonizing the host innate immune response. NW, but not OW, arenavirus Z was shown to bind RIG-I and inhibits IFN-*β* activation [20]. The recently reported NMR structure of LASV Z [17] should facilitate future structure-function studies aimed at the elucidation of the likely several roles played by Z in the arenavirus life cycle.

## 4. The Z Protein Is the Driving Force of Arenavirus Budding

The arenavirus Z protein has been shown to have bona fide budding activity [21–23]. Many enveloped viruses possess a matrix (M) protein that is often the main driving force of viral budding. Accordingly, the sole expression of this M protein can produce virus-like particles (VLPs). Frequently M proteins contain short amino acid motifs, called L (late) domains that play a critical role in virus budding. To date, the sequences PT/SAP, PPXY, and YPXnL (YPXL) have been well established as L domain motifs [24–26]. In addition to these L domains, the FPDL motif and several other short amino acid motifs have also been reported to function as L domains [24–26]. These L domain motifs exert their activity in virus budding by mediating the interaction with specific host cellular factors. Thus, the PT/SAP motif binds to Tsg101, a component of ESCRT-I (endosomal sorting complex required for transport-I) and initiates the budding process [27, 28]. Vps4A/B is AAA-type ATPases involved in catalyzing the disassembly and recycling of the membrane-bound ESCRT complexes [29–31]. Evidence indicates that the M protein of many, but not all, enveloped viruses have the ability to recruit ESCRT complex to their budding sites [24–26]. In addition to M, several other viral proteins, including Sendai virus (SeV) C protein and Marburg virus (MARV) NP, have been found to bind directly to ESCRT proteins, Alix/AIP1, or both and contribute to the budding process [24, 32–35]. Interestingly, some enveloped viruses, including influenza, are able to execute very efficiently the budding process without the ESCRT machinery [24, 36]. It is worth noting that although the M protein of VSV contains both PTAP and PPPY L domain motifs that interact with Tsg101 and Nedd4; respectively, Tsg101 and Vps4A were not required for efficient budding of VSV [37]. Rous sarcoma virus (RSV) Gag has a PPPY motif whose activity has been shown to be regulated by late-domain-interacting protein (LDI-1, Nedd4 chicken homolog) [38, 39]. Recently LYPSL motif in RSV Gag was shown to serve as a second L domain motif [40].  Table 1 summaries the variety of interactions observed between L domains present in M proteins of enveloped viruses and their host cellular interacting partners. 

**Figure 2:**
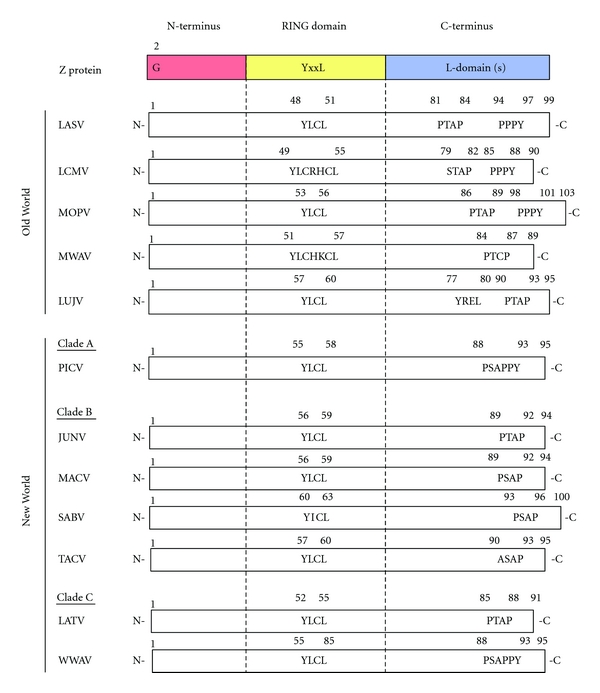
Organization of arenavirus Z protein. All arenavirus Z proteins have G at position 2 (G2). Within the centrally located RING domain, all known arenavirus Z proteins possess an YxxL (or YxxL-like) motif. At their C-terminus, arenavirus Z proteins have different types of L domains. Lassa virus (LASV), lymphocytic choriomeningitis virus (LCMV), Mopeia virus (MOPV), Merino Walk virus (MWAV), Lujo virus (LUJV), Pichinde virus (PICV), Junin virus (JUNV), Machupo virus (MACV), Sabia virus (SABV), Tacaribe virus (TACV), Latino virus (LATV), Whitewater Arroyo virus (WWAV).

 The family Arenaviridae currently includes 23 antigenically related viruses classified into two groups: OW and NW. This classification was originally established based on serological cross-reactivity but is well supported by recent sequence-based phylogenetic studies. OW arenaviruses constitute a single lineage, while NW arenaviruses segregate into clades A, B, and C. Recently, Lujo (LUJV) and Merino Walk (MWAV) viruses were identified as newly identified members of the OW group [41, 42]. Interestingly, among different arenaviruses, there are significant differences in the type of L domain motifs present within their Z proteins (Figure 2). LCMV Z contains a canonical PPPY L domain and the PT/SAP-like domain STAP, whereas the Z of LASV and Mopeia virus (MOPV) which as LCMV are also members of the OW arenavirus group, contain, however, both PTAP and PPPY canonical L domains, but the Z of the also OW member Lujo virus contains only the PTAP L domain. On the other hand, the Z of many of the NW arenaviruses including JUNV, MACV, GATV, and Sabia (SABV) viruses contain the PT/SAP L domain. In addition Z proteins of Pichinde (PICV) and Whitewater Arroyo- (WWAV) viruses contain PTAP and APPY- (PPPY-like-) overlapping L domains, similar to those of Ebola virus (EBOV) VP40 L domain (PTAPPEY). Some arenavirus Z proteins do not contain canonical L domains but rather closely related motifs as in the case of the NW TACV and OW MWAV whose Z proteins contain ASAP and PTCP, respectively, L-like domains. In addition, all known arenavirus Z proteins contain an YxxL motif within the RING domain, but at least for TACV Z protein, it did not influence the Z budding activity [43, 44] (Figure 2). The relative contribution of the different types of L domains to Z-mediated budding appears to be influenced by different factors including the virus species. Thus, for LASV the PPPY L domain appears to have a stronger contribution to budding than the PTAP motif [22, 23]. PPPY motif seems to have critical function compared to PTAP motif. In the case of TACV, the L-like domain ASAP was found to lack budding activity, and TACV Z-mediated budding was also Tsg101-independent but Vps4A/B-dependent [43, 44]. 

In addition to the critical role played by the L domain motifs in Z-mediated budding, glycine at position two (G2) was strictly required for Z-mediated budding. G2 is conserved among all known arenavirus Z proteins (Figure 2) and is required for Z myristoylation and its subsequent targeting of membranes [43, 48, 49]. Accordingly, mutation G2A abrogated Z-mediated budding. Consistent with these findings, treatment with 2-OHM (DL-2-hydroxymyristic acid), an inhibitor of protein myristoylation, caused a dramatic reduction on Z-mediated budding and production of infectious virus progeny [43, 48].

## 5. Novel Strategies to Identify Cellular Factors Contributing to Z-Mediated Budding and Small Molecule Inhibitors of Z-Mediated Budding

As with many other bona fide viral budding proteins, Z-mediated budding requires its interaction with specific cellular factors within the endosomal/multivesicular body pathway as we discuss below. The identification and characterization of LASV-Z-host protein interactions involved in virus budding may uncover novel anti-arenavirus targets, and facilitate the development of screening strategies to identify drugs capable of disrupting viral budding and thereby preventing virus propagation. The ability of Z to direct self-budding in the absence of other viral proteins should facilitate the development of assays amenable to both genetics and chemical High Throughput Screening (HTS) to identify host cellular proteins required for Z-mediated budding, as well as small molecule inhibitors of this process. To this end, the emergence of RNA interference as a pathway that allows the modulation of gene expression has enabled functional genetic screens in mammalian cell types. Likewise, combinatorial chemical libraries have emerged as a leading source of compounds for biological screens, and; therefore, it should be feasible to identify small molecule inhibitors of Z-mediated budding by screening chemical libraries using appropriately designed cell-based assays of Z-mediated budding. In this regard, recent findings have shown that the fusion of the smaller (185 amino acids) luciferase from *Gaussia princeps *(GLuc) to Z resulted in a chimeric protein (Z-GLuc) that retain wild-type Z budding activity that could be monitored by direct measuring of GLuc activity in tissue culture supernatant of Z-GLuc transfected cells. Initial studies have shown that this Z-GLuc-based budding assay consistently exhibits high signal-to-noise ratio (S/N) values (average 10-fold) [50], suggesting that it should be amenable for the development of both genetic and chemical HTS to identify host cellular genes contributing to Z-mediated budding and small molecule inhibitors of Z-mediated budding, respectively.

## 6. Contribution of the ESCRT Machinery to Arenavirus Budding

The ESCRT machinery was originally identified as the Class E subset of vacuolar protein sorting (VPS) genes required for the correct sorting of soluble hydrolases from the yeast Golgi to the vacuole [51–53]. The essential VPS-mediated sorting step occurs during MVB (multi vesicular body) formation, when ubiquitinated proteins and lipids present on the limiting endosomal membrane are recognized and sorted into endosomal membrane microdomains, which ultimately invaginate and form vesicles that bud into the lumen to create the MVB [54–56]. Vesicles of the MVB subsequently fuse with lysosome, thereby exposing the internal vesicles to the degrading lipases and proteases in this organelle. ESCRT pathway is also involved in the membrane abscission event at the conclusion of cell division [57, 58]. ESCRT-I contains Tsg101, Vps37, Vps23, and Mvb12A/B and has been shown to be recruited from the cytoplasm to the surface of maturing endosomes [29, 54–56, 59, 60]. Components of ESCRT-I, especially Tsg101, recognize ubiquitinated protein cargos and interact with ESCRT-II that participates in protein sorting and vesicle formation. It should be noted that Alix/AIP1 was found to bridge ESCRT-I and ESCRT-III using a different way to that used by ESCRT-II [61, 62]. ESCRT-III components also recruit another class E proteins, Vps4A/B, which are AAA-type ATPases involved in catalyzing the disassembly and recycling of the membrane-bound ESCRT complexes [29–31]. Notably, the abscission event during cellular cytokinesis, a process topologically similar to endosomal vesicle formation, utilizes the same ESCRT machinery [29, 58, 63]. 

**Figure 3:**
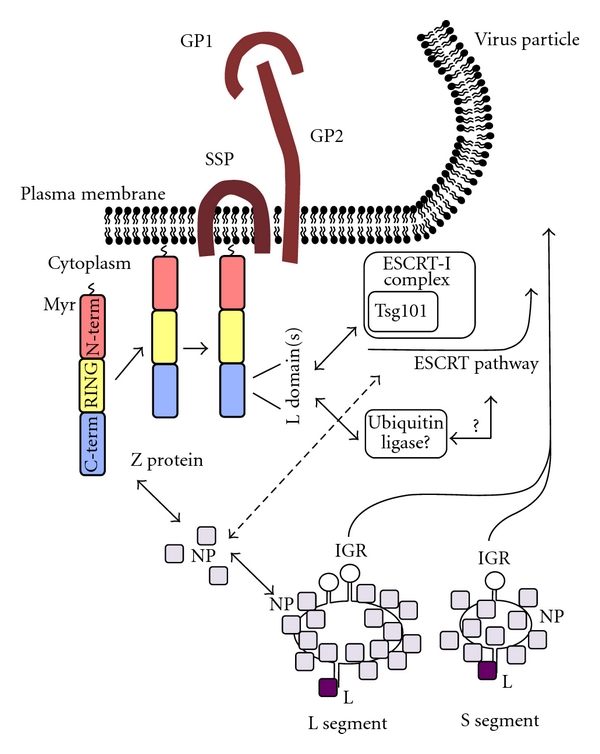
Model of arenavirus budding. Myristoylation of Z at G2 facilitates its interaction with the plasma membrane (PM), where Z likely forms higher-order complexes. L domains located within the C-terminus of Z facilitate its interactions with host cellular factors to allow Z to utilize the ESCRT machinery of the cell for cell egress (budding). SSP, stable signal peptide.

Myristoylation of Z facilitates its attachment to the PM where Z is likely recognized by Tsg101 through the PT/SAP L domain motif present in Z (Figure 3). However, there is not direct biochemical evidence that Z binds to Tsg101 directly. Nevertheless, depletion of Tsg101 by siRNA resulted in decreased levels of both LASV and LCMV Z-mediated budding [21, 23]. Intriguingly, when the ASAP motif present on TACV-Z protein was mutated to AAAA, VLP production levels were not affected, suggesting in contrast to LCMV and LASV that TACV Z-mediated budding does not utilize Tsg101 [43, 44]. However, results of siRNA-mediated depletion and the use of dominant negative mutants indicated that Vps4A/B was necessary for both LASV and TACV Z-mediated budding activity [21, 43]. These results would suggest that the last step within the ESCRT pathway is necessary for both LASV and TACV Z-mediated budding. 

The budding activity of some M proteins has been shown to be increased by the contribution of other viral proteins as illustrated in the case of the nucleoprotein (NP) and glycoprotein (GP) of the EBOV and MARV viruses [64, 65]. However, studies on LCMV and LASV Z-mediated budding failed to uncover a contribution to budding by other viral protein. In contrast, TACV NP was shown to enhance Z-mediated VLP production [44].

Nevertheless, recently published work has shown that Mopeia virus (MOPV) NP incorporation into VLP is mediated by AIP1/Alix via interaction with the YLCL motif present on Z [45, 46]. 

## 7. Tetherin/BST-2/CD317 as an Antagonist of Arenavirus Budding

Tetherin was identified as an IFN-inducible antiviral cellular factor that tether HIV virions at the PM [66, 67]. Subsequently studies have extended these findings to other viruses [68]. As with other host innate immune defense factors, several viruses have evolved mechanisms to counteract tetherin-mediated antiviral activity [69]. Tetherin has been shown to inhibit LASV Z-mediated budding [70]. Accordingly, 293T cells constitutively expressing tetherin resulted in decreased production levels of LASV and MACV virion particle production, whereas siRNA-mediated knockdown of endogenous tetherin in HeLa cells resulted in increased production levels of LASV and MACV virion particle production. These results would suggest that LASV does not possess any tetherin-antagonizing function as described for other viral proteins including HIV-1 Vpu, EBOV GP, and KSHV K5 [66, 71–73]. 

An issue that remains to be investigated relates to the contribution of tetherin to host protection and viral pathogenesis. An attractive hypothesis, but still without experimental support, would be that tetherin could do to some degree slow the process of virus propagation in vivo and thereby facilitating both the action of the host innate immune defense mechanisms and antigen presentation leading to a more robust host adaptive immune response that could control and eliminate the virus.

## 8. Perspectives on Arenavirus Z-Mediated Budding

Current evidence indicates that many enveloped viruses use the ESCRT machinery to exit from the cell. In the case of arenavirus budding, Z-Tsg101 interaction appears to facilitate access of Z to the ESCRT machinery. Despite significant recent progress in defining the basic aspects of arenavirus budding, there are still a large number of issues that have not been investigated including: (1) Identification and functional characterization of host cellular factors that interact with the PPXY L domain motif present in LASV, LCMV, and some other arenavirus Z to facilitate virus budding. PPXY L domain motifs present in EBOV and MARV VP40 have shown to mediate interaction with Nedd4.1 [74–76]. Likewise, for several retroviruses, the interaction of Gag PPXY L domains with ubiquitin ligases has been shown to contribute to the regulation of viral budding [24, 25, 38, 39]. How ubiquitin ligases may regulate budding remains unknown, but recent published data have shown arrestin-related proteins to connect ubiquitin ligases and the ESCRT machinery [77]. It is important to know whether specific ubiquitin ligase may regulate Z-mediated budding, and if so, what are the mechanism underlying this regulation. (2) Ubiquitin or ubiquitin-like molecules (UBLs) have been shown to modify the properties and budding activity of HIV-1 Gag and EBOV VP40 proteins [78]. It is currently unknown whether these protein modifiers may also have a role in the regulation of arenavirus budding. (3) The mechanisms by which arenavirus RNP interacts with Z and GP to form budding mature infectious progeny are largely unknown. (4) Whether the species-specific and type of cell influence arenavirus budding and the biological implications regarding the outcome of infection are issues that have not been investigated. 

Detailed understanding of the virus-host cell protein interactions that direct arenavirus budding may uncover novel targets for the development of antiviral drugs to combat human pathogenic arenaviruses.

## Figures and Tables

**Table 1:** Summary of different matrix (M) protein L domain motifs and cellular-interacting partners. Characterized viral M proteins, accessory proteins, and their L domains are shown. *1: Alix/AIP1 has been shown to connect Z and NP [45, 46]. *2: there is a discrepancy between the groups for Alix/AIP1 and Vps4 necessity for the budding [34, 35, 47].

Virus	M protein	Accessary protein	L domain	Nedd4-like Ubiquitin ligase	Tsg101	Alix/AIP1	Vps4
HIV-1	Gag		PTAP	○	○		○
			YPXnL			○	

RSV	Gag		PPPY	○			○
			YPXnL				

VSV	M		PTAP		x		x
			PPPY	○			

Ebola virus	VP40		PTAP		○		○
			PPPY	○			

Marburg virus	VP40		PPPY	○	○		○
		NP	PSAP		○		

Lassa virus	Z		PTAP		○		○
			PPPY				

LCMV	Z		PPPY		○		○

Tacaribe virus	Z		ASAP		x		○

Mopeia virus	Z		PTAP			○*1
			PPPY			
		NP				

Sendai virus	M		YLDL			○*2	○*2
		C				○*2	

Influenza virus							x
